# PPAR-α agonism improves whole body and muscle mitochondrial fat oxidation, but does not alter intracellular fat concentrations in burn trauma children in a randomized controlled trial

**DOI:** 10.1186/1743-7075-4-9

**Published:** 2007-04-23

**Authors:** Melanie G Cree, Bradley R Newcomer, David N Herndon, Ting Qian, Dayoung Sun, Beatrice Morio, Jennifer J Zwetsloot, G Lynis Dohm, Ricki Y Fram, Ronald P Mlcak, Asle Aarsland, Robert R Wolfe

**Affiliations:** 1 Preventive Medicine and Community Health, University of Texas Medical Branch, Galveston, USA; 2 Surgery, University of Texas Medical Branch, Galveston, USA; 3 Anesthesiology, University of Texas Medical Branch, Galveston, USA; 4 Shriners Hospitals for Children, Galveston, USA; 5 University of Alabama at Birmingham, Birmingham, USA; 6 UMPE- Laboratoire de Nutrition Humain, Clermont-Ferrand, France; 7 Department of Physiology, Brody SOM, Eastern Carolina University, Greenville, USA

## Abstract

**Background:**

Insulin resistance is often associated with increased levels of intracellular triglycerides, diacylglycerol and decreased fat β-oxidation. It was unknown if this relationship was present in patients with acute insulin resistance induced by trauma.

**Methods:**

A double blind placebo controlled trial was conducted in 18 children with severe burn injury. Metabolic studies to assess whole body palmitate oxidation and insulin sensitivity, muscle biopsies for mitochondrial palmitate oxidation, diacylglycerol, fatty acyl Co-A and fatty acyl carnitine concentrations, and magnetic resonance spectroscopy for muscle and liver triglycerides were compared before and after two weeks of placebo or PPAR-α agonist treatment.

**Results:**

Insulin sensitivity and basal whole body palmitate oxidation as measured with an isotope tracer increased significantly (P = 0.003 and P = 0.004, respectively) after PPAR-α agonist treatment compared to placebo. Mitochondrial palmitate oxidation rates in muscle samples increased significantly after PPAR-α treatment (P = 0.002). However, the concentrations of muscle triglyceride, diacylglycerol, fatty acyl CoA, fatty acyl carnitine, and liver triglycerides did not change with either treatment. PKC-θ activation during hyper-insulinemia decreased significantly following PPAR-α treatment.

**Conclusion:**

PPAR-α agonist treatment increases palmitate oxidation and decreases PKC activity along with reduced insulin sensitivity in acute trauma, However, a direct link between these responses cannot be attributed to alterations in intracellular lipid concentrations.

## Background

Significant alterations in both glucose and fat metabolism occur following burn trauma. Hyperglycemia, due to increased hepatic gluconeogenesis and peripheral insulin resistance, is common [1]. Free fatty acid (FFA) cycling is increased up to three fold, and triglyceride (TAG) deposition in the liver is common [2]. Studies in burned animals indicate that mitochondrial number and oxidative capacity are severely reduced following burn, but how these changes relate to in vivo fatty acid oxidation is unclear [3]. Further, the relation between fat metabolism and insulin sensitivity is not well understood in the severely burned population.

Decreased β-oxidation of FFA's and increased circulating concentrations of plasma FFA's are both likely related to insulin resistance. Offspring of type 2 diabetics who appear otherwise healthy have increased intracellular muscle and liver fat and decreased mitochondrial number, size, and fatty acid oxidation rates [4,5]. It has been theorized that a decrease in mitochondrial function, and thus β-oxidation, causes intracellular TAG to accumulate, thereby contributing to the development of insulin resistance [6]. The accumulation of tissue TAG may not only be due to a decrease in the oxidation rate of fatty acids, but also to an increase in fatty acid delivery via plasma TAG and FFA. It is further proposed that intra-cellular TAG per se may not cause insulin resistance, but instead may be associated with increases in TAG metabolites such as diacylglycerol (DAG) and long chain fatty acyl CoA [6]. Both DAG and long chain fatty acyl CoA have been shown to disrupt the insulin signaling pathway at the level of the insulin receptor signalling-1 protein (IRS-1), preventing translocation of glucose transporter to the cell surface membrane, and thus insulin stimulated glucose uptake [6]. This response has been proposed to be mediated by activation of protein kinase C-θ (PKC-θ) and protein kinase C-β (PKC-β) [6].

Peroxisome proliferator activating receptors (PPAR) are nuclear receptors that, when stimulated by endogenous lipids, activate specific genes involved in fat metabolism. Of interest is the apparent ability of PPAR-α agonists to increase fat oxidation and improve insulin sensitivity. Significant decreases in fasting plasma glucose, insulin and TAG, with a concomitant decrease in muscle and liver TAG have been reported in lipoatrophic mice, mice given high fat diets and diabetic Zucker rats after treatment with PPAR-α agonists [7,8]. PPAR-α agonists also decrease intracellular fatty acyl CoA and malonyl CoA, and increase fatty acid oxidation in rodents [9,10]. PPAR-α agonist treatment in human myocytes increased β-oxidation of oleate and decreased oleate incorporation into TAG [11]. Despite the encouraging results in animal and in vitro studies, results of treatment in young and middle aged humans with PPAR-α agonists have not been as well defined [12-14]. Thus, in certain models the PPAR-α agonists affect fat metabolism by increasing mitochondrial oxidative capacity, yet the role in humans is not well established.

We have recently demonstrated that PPAR-α agonist treatment improves both peripheral and hepatic glucose sensitivity and improves the response of the insulin signaling cascade in muscle to insulin in burns [15] but, the effect of PPAR-α agonism on fat metabolism has not been examined. Further, thermal injury in pediatric patients provides unique model to investigate acute onset insulin resistance, since the injuries are similar and quantifiable, and the patients rarely have pre-existing medical conditions. For this reason, we investigated if the PPAR-α agonist fenofibrate could increase palmitate oxidation and decrease intracellular lipids, presumably through increasing mitochondrial fatty acid oxidation in pediatric burn trauma patients.

## Research design and methods

This was a prospective, randomized, double-blind placebo controlled clinical trial. The protocol was approved by the IRB at the University of Texas Medical Branch. A legal guardian provided permission for participation of the child. Assent was obtained in children aged 7–17 when medically possible.

Children aged 4–17 and > 20 kg with > 40% total body surface area burns that would require skin grafting, who arrived at our hospital within 96 hours of injury were eligible. Children with major electrical burns, renal or hepatic failure, iodine allergies, severe sepsis or previous cardiac arrest were excluded.

**Figure 1:**
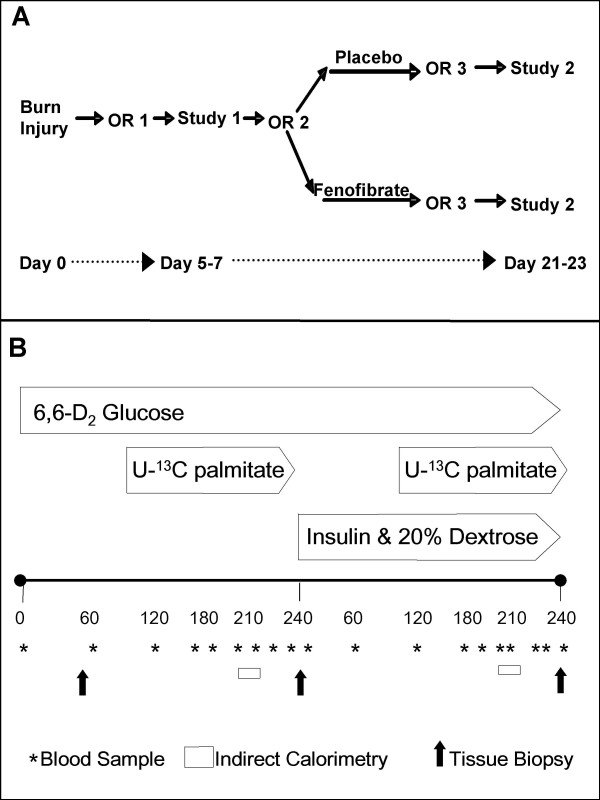
**Overall study design and tracer design**. A) Overall randomization B) Tracer infusion protocol. A four hour basal period was followed a four hour hyperinsulinemic-euglycemic clamp. Glucose isotopes were infused for the duration of the study, and palmitate for the last two hours of each period.

Two 8 hour isotopic tracer studies were conducted ~2 weeks apart (Figure 1). The studies were performed ~4 days after the child's first and third excision and skin auto-grafting operations. This timing avoided acute surgery-induced metabolic changes, yet made use of the existing arterial and venous access. The two week treatment interval was chosen so as to guarantee similar clinical conditions in all patients, since many patients with burns 40–60% may be discharged from intensive care after recovery from their third surgery.

Children were treated between the studies daily with either the PPAR-α agonist fenofibrate (Tricor^©^) or placebo, as pre-determined by a randomization schedule. Five mg/kg of fenofibrate was ground in 1–2 cc of 200 proof ethyl alcohol and suspended in drug suspension agent with flavoring. The placebo consisted of the suspension and flavoring agents. Treating physicians and nurses were blinded to drug allocations.

Each study was preceded by a 4 hour fast with IV fluids of only saline, and 8 hours without blood or albumin transfusions. Background samples were taken from a venous catheter prior to a 4 hour basal period, followed by a 4 hour hyperinsulinemic-euglycemic clamp. During the clamp, insulin was infused at a rate of 1.5 mU/kg/min into a central vein and 20% Dextrose was simultaneously infused to maintain a plasma glucose concentration between 80–90 mg/dL. An infusion of U-^13^C palmitate was started at a rate of 0.08 μmol/min/kg after a 150 μmol/kg NaH^13^CO_3 _prime two hours after the start of each period, and 80 minutes were allowed to reach steady state before four sets of arterial and venous blood samples and breath samples were taken 10 min apart. A V-max 29 metabolic cart (Sensormedics, Yorba Linda, CA) was used during the sampling period to measure the volume of CO_2 _expired. The same primed infusion of U-^13^C palmitate was again started 2 hours after the start of the clamp with an identical sampling schedule. Liver and soleus TG were assessed by magnetic resonance spectroscopy (MRS) as described previously [16].

Medical care was determined by faculty surgeons, fellows and residents according to clinical protocols that have been previously described [17]. Patients were fed with Vivonex T.E.N. ^© ^(Novartis, Minneapolis, Minnesota -82% carbohydrate, 15% protein, 3% fat) at 1.4 times their measured resting energy expenditure. The resting metabolic rate was determined once a week in the early morning at 30°C with a V-max 29 metabolic cart (Sensormedics, Yorba Linda, CA). The patients received nutritional supplements including a multi-vitamin, folic acid, zinc, and vitamin C.

Plasma FFA's were isolated as described previously for analysis of concentration and enrichment [18]. The isolated FFA solid was transferred into FAME and reconstituted in heptane for GC and MS. Enrichment was measured with an HP-6890 GC/Agilent 5973 Mass Spectrometer (Wilmington, DE) for the ions of [M+0]^+^, [M+15]^+ ^and [M+16]^+ ^at m/z 270, 285 and 286, corresponding to the molecular ions of palmitate and its isotopic tracers. Concentrations were measured on a HP- 5890 GC-FID with 3392A integrator or a HP-6890 GC-FID (Agilent technologies, Palo Alto, CA), using heptadecanoic acid as an internal standard.

Blood CO_2 _samples were collected in 15 ml vacutainer tubes containing 85% phosphoric acid and breath CO_2 _samples were collected in plain 15 ml vacutainer tubes. Samples were analyzed with IRMS (VG ISOGAS, Cheshire, England) and a standard curve prepared from the phosphoric acid and 45 CO_2 _standard (Bayer, East Walpole, MA) was used to calculate the blood CO_2 _concentration.

Plasma glucose concentration was measured on an YSI 2300 Stat glucose/lactate analyzer (YSI, Inc. Yellow Springs, OH). The isotopic enrichment of the penta-acetate derivative of plasma glucose was determined by GC/MS as described previously [19]. Serum insulin concentrations were measured using radioactive immuno assay (Diagnostic Laboratories, Los Angeles, CA).

Liver fat (LFAT) and muscle triglycerides (IMCL) were measured using magnetic resonance spectroscopy as described previously [16], except that the settings of the scans were altered to decrease the amount of time in the machine for the patients. For the liver scans, 128 scans were performed, rather than 256, and a repetition time 1188 ms was used rather than 2000 ms. For the IMCL scans, the repetition time was also decreased to 1188 ms. Muscle DAG concentrations were measured as previously described [20]. Fatty acyl-carnitine and CoA concentrations were measured in muscle tissue as previously described [21,22].

Mitochondrial enzyme activity of β-hydroxyacetyl buterate (β-HAD) was measured from homogenates of vastus lateralis biopsies in a sucrose/EDTA/Tris buffer as previously described [23]. Maximal mitochondrial coupled and uncoupled oxidation of palmitate in the presence of excess carnitine were measured in permeabilized skinned muscle fibers from fresh muscle tissue as previously described [24]. Following these measurements, the samples were weighed, and the citrate synthase activity and protein contents were measured [25,26].

The concentration of membrane bound, or activated PKC-θ and PKC-β were measured from frozen muscle samples using Western-blot separation and antibody probing methods [27].

### Calculations

The rate of endogenous palmitate release by adipocytes (appearance into plasma) was calculated by measuring the dilution of the infused tracer by unlabeled palmitate. The calculation was:

Rate of appearance=tracer infusionarterial enrichment
 MathType@MTEF@5@5@+=feaafiart1ev1aaatCvAUfKttLearuWrP9MDH5MBPbIqV92AaeXatLxBI9gBaebbnrfifHhDYfgasaacH8akY=wiFfYdH8Gipec8Eeeu0xXdbba9frFj0=OqFfea0dXdd9vqai=hGuQ8kuc9pgc9s8qqaq=dirpe0xb9q8qiLsFr0=vr0=vr0dc8meaabaqaciaacaGaaeqabaqabeGadaaakeaacqqGsbGucqqGHbqycqqG0baDcqqGLbqzcqqGGaaicqqGVbWBcqqGMbGzcqqGGaaicqqGHbqycqqGWbaCcqqGWbaCcqqGLbqzcqqGHbqycqqGYbGCcqqGHbqycqqGUbGBcqqGJbWycqqGLbqzcqGH9aqpdaWcaaqaaiabbsha0jabbkhaYjabbggaHjabbogaJjabbwgaLjabbkhaYjabbccaGiabbMgaPjabb6gaUjabbAgaMjabbwha1jabbohaZjabbMgaPjabb+gaVjabb6gaUbqaaiabbggaHjabbkhaYjabbsha0jabbwgaLjabbkhaYjabbMgaPjabbggaHjabbYgaSjabbccaGiabbwgaLjabb6gaUjabbkhaYjabbMgaPjabbogaJjabbIgaOjabb2gaTjabbwgaLjabb6gaUjabbsha0baaaaa@71EA@

where arterial enrichment is expressed as tracer/tracee ratio and palmitate tracer infusion rate as μmol/kg/min.

To calculate the rate of whole body fat oxidation, the following equation was used:

Palmitate Oxidation=Enrichment CO2×Volume CO2Precursor Enrich×Acetate Corr×(%M+16×16+%M+15×15)
 MathType@MTEF@5@5@+=feaafiart1ev1aaatCvAUfKttLearuWrP9MDH5MBPbIqV92AaeXatLxBI9gBaebbnrfifHhDYfgasaacH8akY=wiFfYdH8Gipec8Eeeu0xXdbba9frFj0=OqFfea0dXdd9vqai=hGuQ8kuc9pgc9s8qqaq=dirpe0xb9q8qiLsFr0=vr0=vr0dc8meaabaqaciaacaGaaeqabaqabeGadaaakeaacqqGqbaucqqGHbqycqqGSbaBcqqGTbqBcqqGPbqAcqqG0baDcqqGHbqycqqG0baDcqqGLbqzcqqGGaaicqqGpbWtcqqG4baEcqqGPbqAcqqGKbazcqqGHbqycqqG0baDcqqGPbqAcqqGVbWBcqqGUbGBcqGH9aqpdaWcaaqaaiabbweafjabb6gaUjabbkhaYjabbMgaPjabbogaJjabbIgaOjabb2gaTjabbwgaLjabb6gaUjabbsha0jabbccaGiabboeadjabb+eapnaaBaaaleaacqaIYaGmaeqaaOGaey41aqRaeeOvayLaee4Ba8MaeeiBaWMaeeyDauNaeeyBa0MaeeyzauMaeeiiaaIaee4qamKaee4ta80aaSbaaSqaaiabikdaYaqabaaakeaacqqGqbaucqqGYbGCcqqGLbqzcqqGJbWycqqG1bqDcqqGYbGCcqqGZbWCcqqGVbWBcqqGYbGCcqqGGaaicqqGfbqrcqqGUbGBcqqGYbGCcqqGPbqAcqqGJbWycqqGObaAcqGHxdaTcqqGbbqqcqqGJbWycqqGLbqzcqqG0baDcqqGHbqycqqG0baDcqqGLbqzcqqGGaaicqqGdbWqcqqGVbWBcqqGYbGCcqqGYbGCcqGHxdaTcqGGOaakcqGGLaqjcqqGnbqtcqGHRaWkcqaIXaqmcqaI2aGncqGHxdaTcqaIXaqmcqaI2aGncqGHRaWkcqGGLaqjcqqGnbqtcqGHRaWkcqaIXaqmcqaI1aqncqGHxdaTcqaIXaqmcqaI1aqncqGGPaqkaaaaaa@A2D2@

The precursor enrichment measurement was made prior to the infusion of the tracer and the acetate correction factor used for these calculations was 0.90, as determined by studies in a similar patient population of pediatric burns using 1,2 C^13^-Acetate [28]. %M+16 and %M+15 are the percentages of M+16 and M+15 in the precursor pool.

Glucose infusion rate in mg/kg/min was calculated by measuring the amount of 20% dextrose infused to maintain plasma glucose between 80–90 mg/dL and corrected for the actual concentration of dextrose (16–19%).

### Statistics

All data are presented as the mean ± standard error of the mean. Results within each group were compared between pre and post-treatment with a two-tail paired students t-test. The basal and clamp periods were also compared in this manner. The change from the pre-to post treatment was compared between the two treatment groups with a one tailed unpaired t-test. All statistics were preformed with Sigma Stat software package, version 2.03.

## Results

9 children in each group completed the study. The mean age was 8 ± 1 years in the placebo (PLA) and 6 ± 1 years in fenofibrate (FEN). The groups were similar in terms of gender and race distribution, with the majority of patients Hispanic males. The burn size was approximately 65% in both groups, and the percent third degree burn was similar between groups, and above 40%. Detailed clinical data have been presented previously [15], but there were no significant hepatic or renal abnormalities, and both groups were similar at baseline. Plasma lipids were unchanged with treatment in either group (Table 1). Plasma cortisol, TNF-α and IL1 and Il-6 were unchanged by either treatment [15].

**Table 1:** Plasma Lipids

	**PLA**		**FEN**	
**Measurement**	**Pre**	**Post**	**Pre**	**Post**

Cholesterol	92 ± 8	100 ± 5	73 ± 8	92 ± 6
Triglycerides	163 ± 32	116 ± 9	121 ± 8	168 ± 16
HDL	14 ± 8	20 ± 2	8 ± 1	14 ± 2
LDL	46 ± 8	56 ± 4	43 ± 8	47 ± 6
VLDL	33 ± 6	23 ± 2	24 ± 2	36 ± 4

Plasma lipids Measured before and after placebo or fenofibrate treatment. There were no significant changes with treatment in any of the plasma lipid measured in either group.

We have previously shown that fenofibrate treatment significantly increases insulin stimulated glucose uptake by 59 ± 11% compared to 16 ± 19% in placebo, and that the children have insulin sensitivity that is 50 to 75% lower than expected [15]. Further, fenofibrate may increase hepatic suppression of glucose release in response to insulin [15]. The rate of whole body fat oxidation pretreatment was similar between the groups (Table 2). During hyper-insulinemia, fat oxidation was suppressed to approximately 40% of basal values. Post-treatment, the basal rate of palmitate oxidation in PLA was unchanged, whereas there was a significant (P = 0.004) increase in FEN. During hyper-insulinemia post-treatment, oxidation decreased in a similar proportion as pre-treatment. The percent of palmitate taken up by tissues and subsequently oxidized was the same at pre-treatment in both groups during basal and clamp periods. After treatment the percentage of palmitate oxidized during the basal states increased substantially in FEN, but not PLA (P = 0.04)(Table 2).

**Table 2:** Palmitate kinetics and Protein Kinase-C activation

	**PLA**		**FEN**	
**Measurement**	**Pre**	**Post**	**Pre**	**Post**

**Palmitate oxidation**				
Basal	0.85 ± 0.19	1.03 ± 0.12	0.99 ± 0.13	1.40 ± 0.13*
Clamp	0.32 ± 0.05†	0.44 ± 0.04†*	0.35 ± 0.05†	0.74 ± 0.13†*
**Percent uptake oxidized**				
Basal	28 ± 3	28 ± 3	24 ± 2	30 ± 2*
Clamp	27 ± 3	28 ± 1	27 ± 3	37 ± 5
**Palmitate Release**				
Basal	2.96 ± 0.5	3.66 ± 0.3	4.06 ± 0.4	4.7 ± 0.3
Clamp	1.20 ± 0.2†	1.94 ± 0.2†	1.15 ± 0.2†	2.04 ± 0.3†
**PKC-β**				
Basal	0.35 ± 0.10	0.30 ± 0.11	0.25 ± 0.04	0.70 ± 0.25
Clamp	1.12 ± 0.60	1.64 ± 0.70	1.99 ± 0.58	1.64 ± 0.46
**PKC-θ**				
Basal	1.38 ± 0.27	1.15 ± 0.36	0.87 ± 0.28	1.26 ± 0.38
Clamp	0.98 ± 0.17	1.15 ± 0.48	0.87 ± 0.31	0.33 ± 0.11*

The rate of palmitate oxidation is shown in (μmol/kg/min), percent uptake oxidation and whole body palmitate release (μmol/kg/min). * indicates a change from pre to post treatment, and † represents a change from basal to clamp. Of interest was a significant increases in the rate of palmitate oxidation in the basal (P = 0.004) and clamp (P = 0.03) states following fenofibrate treatment, and the percent uptake oxidized (P = 0.04) Protein Kinase-C β membrane translocation is shown in relative units, representing activation in both the basal and the clamp hyper-insulinemic state following fenofibrate treatment. Protein Kinase-C θ membrane translocation, representing activation representing activation in both the basal and the clamp hyper-insulinemic state following fenofibrate treatment. * The activation of PKC-θ decreased significantly during hyper-insulinemia following fenofibrate treatment (P = 0.004).

The systemic release of FFA in the body was quantified as the appearance of palmitate and was similar in both groups pre-treatment. Palmitate appearance was not significantly suppressed during hyper-insulinemia (Table 2). Post-treatment the release of palmitate in both groups was similar to the respective pre-treatment values, and again the suppression of FFA appearance during hyper-insulinemia was not significant.

Pre-treatment, PKC-β activity tended to increase during the clamp compared to the basal state in both treatment groups (Table 2). After treatment- the response of PKC-β activation was the same. PKC-θ activity did not respond to insulin pre-treatment. In PLA after treatment, the basal activity of PKC-θ trended to increase and there was no difference between the clamp and basal states after treatment. The basal state of PKC-θ in FEN post-treatment was similar to that seen in the other groups. However, following the insulin infusion, the level of PKC-θ was significantly decreased compared to the pre-treatment clamp activity (P = 0.004).

Liver fat was assessed by MRS in 7 of 9 children in each group. There was no change in LFAT or soleus IMCL after treatment in either group (Table 3) Total (16:0, 18:0, 18:1 and 18:2) DAG and total long chain fatty acyl CoA from muscle biopsy samples were also unaltered with treatment (Table 3) In DAG, the predominate species was 18:0 (32–46% of total), whereas 18:1 was the predominate species in both fatty Acyl CoA (46–50% of total) and Carnitine (40–43% of total).

**Table 3:** 

	**PLA**		**FEN**	
	
**Measurement**	**Pre**	**Post**	**Pre**	**Post**
**LFAT **(% LFAT/control)	26 ± 9	27 ± 9	24 ± 7	30 ± 9
**IMCL **(% IMCL/control)	27 ± 8	18 ± 7	30 ± 16	22 ± 9
**Total DAG **(pmol/mg)	482 ± 92	380 ± 59	341 ± 102	357 ± 75
**Total Long Chain fatty acyl CoA **(pmol/mg)	6.0 ± 1.1	5.0 ± 0.8	5.4 ± 0.5	6.4 ± 1.1
**Total Long chain fatty Acyl carnitine **(pmol/mg)	55 ± 18	62 ± 18	56 ± 13	58 ± 19
**Percent of CoA/Carnitine**	10 ± 6	8 ± 4	9 ± 4	10 ± 5

Basal levels of intracellular triglyceride percentages for the liver and soleus muscle are shown, and did not change with treatment in either group. Basal concentrations of total long chain species (16:0, 16:1, 18:0, 18:1 and 18:2) of DAG, fatty acyl CoA and fatty acyl carnitine also did not change with either treatment.

The mitochondrial state 3 oxidative respiration of palmitate, which is coupled with ADP use, was increased following treatment in FEN (P = 0.03) and decreased following treatment in PLA (P = 0.02) (Table 4). The state 4 uncoupled oxidative rate did not change with either treatment, although there was trend for increase in PLA, and thus the ratio of state 3 to state 4 oxidation, or respiratory control ratio, increased significantly in FEN, compared to PLA (Table 4). The increased respiratory control ratio, reflective of an increased proportion of coupled oxidation, could not be explained by a change in the activity level of β-HAD, a key mitochondrial enzyme in FFA oxidation, which did not significantly change from pre to post treatment in either the PLA or the FEN groups (Table 3).

**Table 4:** Mitochondrial oxidation and enzyme levels

	**PLA**		**FEN**	
	
**Measurement**	**Pre**	**Post**	**Pre**	**Post**
**State 3 oxidation **(μmol O_2_/mg CS/min)	0.87 ± 0.05	0.73 ± 0.07*	0.80 ± 0.10	1.17 ± 0.12*
**State 4 oxidation **(μmol O_2_/mg CS/min)	0.58 ± 0.04	0.73 ± 0.13	0.60 ± 0.06	0.60 ± 0.07
**Ratio of State3/State4**	1.51 ± 0.11	1.30 ± 0.31	1.40 ± 0.17	2.01 ± 0.16*
**β-HAD **(μmol substrate/g wet tissue/min)	5.0 ± 0.9	4.3 ± 1.4	4.0 ± 0.8	4.5 ± 0.8

State 3 respiration of palmitate in (μmol 02/CS mg protein/min) improved significantly (P = 0.03) after treatment in FEN, whereas there was a significant decrease seen in PLA (P = 0.02) State 4 oxidation of palmitate in (μmol 02/CS mg protein/min) did not change with either treatment. The ratio of state 3 to state 4 palmitate oxidation improved significantly (P = 0.02) following fenofibrate treatment. The concentration of β-HAD did not change in either group with treatment. * indicates significant change from Pre to post treatment.

## Discussion

Whole body palmitate oxidation was increased significantly following PPAR-α agonist treatment in the basal state when compared to placebo, as was the percentage of palmitate oxidized. The improvement at the whole body level was reflected by increased muscle mitochondrial capacity for palmate oxidation, but these changes were not due to an increase in β-HAD, and based on the CoA to carnitine ratio, were not likely due to change in fatty acid entry into the mitochondria. These changes in fat oxidation were accompanied by decreased PKC-θ activation, yet the basal concentrations of proposed upstream signaling molecules such as TAG, DAG or fatty acyl CoA were not significantly changed with fenofibrate treatment. Further, plasma lipid concentrations were unaffected by fenofibrate treatment.

While this finding of PPAR-α induced increase in palmitate oxidation is unique in humans, similar findings have been found in animal studies [29]. Further, a very potent PPAR-α agonist was shown to increase palmitate oxidation in human hepatic and muscle cells in a dose dependant manner [30]. No prior studies have measured the effect of PPAR-α agonist treatment in burn patients.

The LFAT measurements in these burned children were similar to those that we have previously found in elderly subjects with insulin resistance [16]. While we have not measured LFAT in healthy children, it can be assumed that this would be low and similar to concentrations in healthy young adults. If this is true, measured LFAT was increased 3 to 4 fold in children at week 1 post-burn, and remained elevated regardless of fenofibrate treatment. It is possible the LFAT was elevated as soon as the first study, as LFAT in pigs doubled four days post-burn [31]. Fenofibrate may not have decreased LFAT because of the short duration of treatment. Animal studies that demonstrated decreased hepatic TAG's after PPAR-α agonist treatment generally have used much larger doses for longer periods of time [7-10]. Furthermore, the endogenous expression of the PPAR-α receptor is much higher in animals than in humans [32]. Thus, while we were unable to detect an effect of PPAR-α of LFAT in this population, perhaps increased doses would affect a metabolic change.

Levels of IMCL were similar to those seen in elderly adults and did not change in either treatment group [16]. Further, the lack of change in the presence of increased palmitate oxidation indicates that increases in IMCL levels may not be due only to decreased oxidation of intracellular lipids. IMCL levels may be increased secondary to adipocyte dysfunction and this may account for the relationship noted in patients with increased adiposity, insulin resistance and increased IMCL [33].

Some studies have found close associations between intracellular lipids such as TAG, DAG and fatty acyl-CoA and insulin sensitivity [6]. However, we did not find these associations in burned children. Basal concentrations of DAG, TAG, fatty acyl-CoA and fatty acyl carnitine did not decrease significantly as insulin sensitivity increased following PPAR-α treatment in the fenofibrate treatment group. Whereas there may be a Type II error with the variability of the data prior to treatment, the lack of change within each treatment groups, especially the fenofibrate group indicates that there was not a change in DAG concentrations with treatment. Similar results were reported in rats fed a high fat diet that had over expression of hepatic malonyl CoA dehydrogenase [34]. Malonyl CoA dehydrogenase breaks down malonyl CoA and presumably increases β-oxidation of fats. Muscle, fat and whole body insulin sensitivity improved in these animals but IMCL did not change, and there was a trend for increased fatty acyl CoA production. Furthermore, rats fed a high fat diet and treated with Wy-14643, a potent PPAR-α agonist, demonstrated an increase in long chain fatty acyl-CoA's and insulin mediated glucose uptake, and decreased intramuscular TAG [35]. A similar study found that chronic WY-14643 administration in rats did not lower palmitoyl CoA [36]. Four weeks of treatment in overweight, diabetic ob/ob rats, with WY-16643, had no effect on palmitoyl CoA in muscle, and increased oleoyl CoA more than ten-fold in skeletal muscle [37]. These conflicting results indicate that the association between intracellular lipids and insulin sensitivity is perhaps not as clear as previously thought. The fact that the fatty acid distribution in DAG differs from that in other intracellular lipids, which suggests that there is not a straightforward precursor-product relation as previously suggested [33] further complicates the potential link between altered intracellular lipids, insulin signaling and insulin sensitivity.

It is possible that the increase in mitochondrial oxidation was due to changes in the respiratory chain, rather than at other regulatory sites within fatty acid metabolism. We have previously documented that PPAR-α agonism increases pyruvate oxidation, and with similar results found with a palmitate substrate, the site of increased metabolism would need to be regulatory site common to both, i.e. either the TCA cycle or the respiratory chain [15]. Further, if fatty acyl CoA is rapidly broken down within the mitochondria, then the ratio of fatty acyl CoA to fatty acyl carnitine can be used as a rough estimate of carnitine palmitate transferase-1 (CPT-1) activity. We found that there was substantially more fatty acyl carnitine than CoA, perhaps indicating that CPT-1 was not inhibited. Further, since the mitochondrial oxidation of palmitate and pyruvate are similar, and pyruvate metabolism is not affected by CPT-1, it is unlikely that this is the site of PPAR-α activity.

PKC-β and PKC-θ are both found in skeletal muscle and the activities vary in animals and humans, and thus we studied both [38]. The levels of PKC-β increased during insulin stimulation in all of the groups, yet increased the most in the PLA groups after treatment. Insulin did not affect PKC-θ pre-treatment, or in the PLA post-treatment, yet there was a significant decrease in PKC-θ in FEN after treatment. No studies to date have looked at the effect of PPAR-α agonists on PKC activity. PKC has been associated with insulin resistance in many settings. PKCs are thought to be inhibitory to glucose transporter translocation by causing serine phosphorylation of the insulin receptor and IRS-1 [6]. In animals overfed with either fat or glucose, increased levels of activated PKC and insulin resistance have been found [39,40]. Increased levels of activated PKC have also been found in diabetic patients [41]. Thus, the reduction in PKC activity may be associated with improved insulin sensitivity. However, the increase in PKC with insulin resistance is typically associated with increased concentrations of DAG, and we did not observe this relationship between PKC and basal concentrations of DAG.

## Conclusion

Fat metabolism in burn patients is altered by PPAR-α agonist treatment. Whole body and mitochondrial oxidation of palmitate were increased following treatment, compared to placebo. Further, PKC activity changed with fenofibrate treatment. However, fenofibrate did not lower the basal concentrations of either liver fat, muscle TAG, DAG, fatty acyl carnitine or fatty acyl-CoA. It may be that the mechanism of insulin resistance post-trauma differs from that seen in diabetes.

## Abbreviations

β-HAD – β-hydroxyacetyl buterate

CPT-1 – Carnitine Palmayl Transferase -1

DAG – Diacylglycerol

FEN – Fenofibrate treated group

FFA – Free fatty acids

IMCL – Intramuscular Lipid Content

IRB – Institutional Review Board

IRS-1 – Insulin Receptor Substrate 1

LFAT – Liver fat

MRS – magnetic Resonance Spectroscopy

PKC – Protein Kinase C

PLA – Placebo treated group

PPAR – Peroxisome proliferator activating receptors

TAG – Triacylglycerol

## Competing interests

The author(s) declare that they have no competing interests.

## Authors' contributions

All authors read and approved the final manuscript.

MGC – Obtained IRB, FDA approval, helped obtain primary grant, conducted all patient studies, analyzed glucose data, performed statistics, wrote manuscript

BRN- Helped adapt MRS procedure for burns, analysis of MRS, edited manuscript

DNH-Obtained IRB approval, provided secondary funding, supervising physician, edited manuscript

TQ-Analyzed mitochondrial oxidation data, edited manuscript

DS- Analysis of Fatty Acyl CoA and Carnitine Samples, DAG method development, edited manuscript

BM-Analyzed mitochondrial enzyme data, edited manuscript

JJZ-Performed insulin signaling analysis, edited manuscript

GLD- Supervised insulin signaling analysis, edited manuscript

RYF- Assisted with patient studies, analyzed glucose data, edited manuscript

RPM – Performed REE and measured CO2 exhaled volume, edited manuscript

AA-Obtained IRB, FDA approval, helped obtain primary grant, safety monitoring physician, edited manuscript

RRW-Obtained IRB, FDA approval, wrote primary grant, supervised all data analyses, wrote manuscript
